# The effect of scutellaria baicalensis and its active ingredients on major depressive disorder: a systematic review and meta-analysis of literature in pre-clinical research

**DOI:** 10.3389/fphar.2024.1313871

**Published:** 2024-03-20

**Authors:** Ying Ma, Xun Zhou, Feng Zhang, Cuiyun Huang, Hong Yang, Wansheng Chen, Xia Tao

**Affiliations:** ^1^ Department of Pharmacy, Changzheng Hospital, Second Military Medical University, Shanghai, China; ^2^ Institute of Chinese Materia Madica, Shanghai University of Traditional Chinese Medicine, Shanghai, China

**Keywords:** scutellaria baicalensis, depression, preclinical study, meta analysis, systematic review

## Abstract

**Background:** Scutellaria baicalensis, the dry root of *scutellaria baicalensis* georgi, is a traditional Chinese medicine with long. In clinic, scutellaria baicalensis is commonly used in prescription for the treatment of depression. Additionally, numerous pre-clinical studies have shown that Scutellaria baicalensis and its active constituents are effective for depression. In this study, we aims to systematically review the roles of scutellaria baicalensis in depression and summarize the possible mechanism.

**Methods:** A systematic review and meta-analysis were conducted to analyze the existing studies on the effects of scutellaria baicalensis on depression in animal models. Briefly, we searched electronic databases including Pubmed and Embase for preclinical trial studies from inception to September 2023. The items in each study were evaluated by two independent reviewers, and meta-analyses were performed on scutellaria baicalensis-induced behavioral changes in the study. Finally, random effects model is used to collect data.

**Results:** A total of 49 studies were identified, and 13 studies were included in the final analysis. They all reported the different antidepressant effects of scutellaria baicalensis and the underlying biological mechanisms. Among the included 13 studies, the results of eight articles SPT[SMD = −2.80, 95%CI(-4.03, -1.57), *p* < 0.01], the results of the nine articles OFT[SMD = −2.38, 95%CI(-3.53, -1.23), *p* < 0.01], and the results of two articles NSFT[SMD = −2.98, 95%CI(-3.94, -2.02), *p* < 0.01] were significantly different from the control group. The risk of bias was moderate in all studies, however, there was a significant heterogeneity among studies.

**Conclusion:** These results preliminarily suggest that scutellaria baicalensis can alleviate depressive behaviors and modulate underlying mechanisms, which is expected to be a promising antidepressant.

## 1 Introduction

Depression (major depressive disorder), a common mental disease, is manifested by persistent feeling of sadness and loss of interest. This kind of disease can disturb person’s ability to work, study, sleep, eat, and enjoy once pleasurable activities. What’s more, people with depression possess cognitive behavioral, social dysfunction, and even suicide tendency in severe cases (Vos et al., 2017; Dwyer Jennifer et al., 2020). In recent years, its commonly defined that depression is global burden of disease with high mortality and morbidity, and high disability rate. In America, In 2017, the World Health Organization revealed that there were more than 300 million depression patients in the world, accounting for about 4.4% of the global population (World Health Organization WHO, 2017). Depression accounts for a large share of the global disease burden, with approximately 264 million people globally estimated to suffer from the condition (World Health Organization (WHO)). In China, a cross-sectional epidemiological study from 2013.07 to 2015.03 revealed that the economic burden of China is about 2.5 trillion US dollars, accounting for 10% of the total global disease burden (Jin et al., 2021). A cross-national comparison reported that lifetime prevalence estimates of major depressive disorders ranged from 1.0% (Czech Republic) to 16.9% (United States), with midpoints at 8.3% (Canada) and 9.0% (Chile), while the 12-month prevalence estimates ranged from 0.3% (Czech Republic) to 10% (United States), with midpoints at 4.5% (Mexico) and 5.2% (West Germany) (Kessler Ronald and Bromet Evelyn, 2013). Another cross-sectional survey analysis conducted in USA also pointed that individuals with depression diagnosis have substantial humanistic and economic burden (Jain et al., 2022). In 2015, The World Health Organization ranked depression as the single largest contributor to global disability, accounting for 7.5% of all years lived with disability (World Health Organization WHO, 2017). These epidemiological studies highlight that depressive disorder is a current issue for public health and will be a future challenge.

More and more studies on depression have shown that numerous factors, such as age, genetics, biology, and environment, influence depression morbidity and mortality (Hammen, 2018; Barrenetxea et al., 2022; Mars et al., 2022). Koh et al. reported that the incidence of population (70–80 years) was higher when compared with the population (60–69) (Barrenetxea et al., 2022). Bai et al. also revealed that in the last 3 decades, the incidence rate of depression among older individuals has increased though the age-standard incidence rate of depression has declined in China (Bai et al., 2022). In addition, the impact of other diseases such as cardiovascular diseases, obesity, and hypertension can not be ignored in recent years (Li et al., 2022). Indeed, there is a bidirectional association between cardiovascular diseases (Bobo et al., 2020), obesity (Luppino et al., 2010), hypertension (Jokela et al., 2014) and anxiety. In Korea, Park et al. found that depression increased the risk of ischemic heart disease by 38% and cerebrovascular disease by 46% among older adults through retrospective cohort study (Park et al., 2020). In addition, people with cardiovascular disease have a significantly increased risk of depression (Lesman-Leegte et al., 2009). An overview of a meta-analysis showed that Obese adults were 55 percent more likely to be depressed, and depressed adults were 58 percent more likely to be obese (Luppino et al., 2010). Lu et al. reported that incidence of depression in China were more high in women than that in men, unemployed people than employed, and those who were separated, widowed, or divorced than people who were married or cohabiting (Jin et al., 2021).

Presently, the clinical treatment strategy for depression contains first-line antidepressant drugs, cognitive-behavioral therapy, and physiotherapy (Kverno and Mangano, 2021). And the first-line antidepressant drugs includes selective serotonin reuptake inhibitor (SSRIs, fluoxetine, paroxetine, and sertraline), serotonin and noradrenaline reuptake inhibitor (SNRIs, venlafaxine, and duloxetine), noradrenergic and specific serotonergic antidepressants (NaSSA, mirtazapine), serotonin receptor antagonists and reuptake inhibitors (SARIs, trazodone), monoamine oxidase inhibitor (MAOI, moclobemide), and tricyclic antidepressants (TCA, imipramine) (Plenge et al., 2021). What’s more, ketamine (Nikayin et al., 2022) and nitrous oxide (Quach et al., 2022) are also used for the resistant depression. However, the cure rate of first-line antidepressants is low, and the adverse reactions of these drugs are obvious, and a response to conventional antidepressants requires several weeks of treatment and carries a non-negligible risk of suicide. Therefore, there is a major medical need for novel and improved antidepressant treatments. Acupuncture and herbal medicine were also used for the treatment of depression, and herbal medicine were shown to had superior efficacy and safety profiles (Chen and Shan, 2019).

*Scutellaria baicalensis* georgi is a herbal medicine frequently used in China, and its dry root (common name: Huang-Qin in Chinese) is widely used in prescription for the treatment of depression (Zhang et al., 2015; Lee et al., 2017). The beneficial effects of the root are due to different bioactive compounds in the brain, some of which are able to cross the blood-brain barrier (BBB). As far as it concerns scutellaria baicalensis, this corresponds to the two main flavonoids, namely, baicalin and baicalein, being purified from the plant’s dry roots (Wang et al., 2018; Zhao et al., 2019). Previous studies have shown that scutellaria baicalensis has a wide range of pharmacological effects including anti-inflammatory, anti-oxidative, neuroprotective, antibacterial, and anti-tumor activities (Zhao et al., 2016; Zhou et al., 2016; Zhao Yikai et al., 2018; Yoon et al., 2020). It has been found that Scutellaria baicalensis and its main components baicalin and baicalein have significant anti-depression effects and mechanism involves many aspects, such as improving the level of monoamine transmitter brain neurotrophic factor, regulating the HPA axis, anti-inflammation, anti-oxidation and promoting neurogenesis (Hai-Yang et al., 2016; Pazini Francis et al., 2017). In addition, as a traditional medicine, Scutellaria baicalensis has produced neuroprotective effects in various models of Parkinson’s disease (Mu X. et al., 2011), Alzheimer’s disease (Zhao J. et al., 2018)and so on. Recent studies have shown that baicalin and baicalein, in addition to protecting dopaminergic neurons from mitochondrial and oxidation-related toxicity, may also have a beneficial effect on DA-related brain diseases by increasing DA levels in the brain (Im H. I. et al., 2005; Zhao J. et al., 2018).

At present, accumulating evidence from the pharmacological effect indicated that scutellaria baicalensis may have great potential in treating depression. Nevertheless, up to now, the pre-clinical studies on scutellaria baicalensis for depression have not been systematically evaluated and summarized. In this study, we conducted a rigorous and comprehensive systematic review and meta-analysis of recent literature on the treatment of depression model animals by scutellaria baicalensis, and explored different behavioral changes and potential mechanisms, aiming to provide evidence and guidance for clinical practice.

## 2 Materials and methods

### 2.1 Search strategies

We searched relevant databases, including PubMed, Web of Science, Embase, and CNKI from inception to September 2023. The main search terms were composed by “Scutellaria baicalensis” or Radix Scutellariae [tiab] OR Scutellaria [tiab] OR baicalin [Mesh] OR baicalein [Mesh] AND (Depression [Mesh] OR “Depressive disorder” [Mesh] OR Depress [tiab] OR “emotional disorder” [tiab] OR “psychological disorder” [tiab] OR “psychological distress” [tiab] OR “emotional distress” [tiab] OR “emotional stress" [tiab]. Subsequently, the two researchers (Ying Ma and Xun Zhou) independently reviewed the title/abstract related to the topic. A full-text read was also performed to find the potential documents that met the eligibility criteria. Importantly, any disagreements between the two researchers were resolved through negotiation or thirdparty consensus.

### 2.2 Inclusion and exclusion criteria

Studies were be included when they meet the following criteria: (1) *in vivo* studies on animal subjects; (2) the animal disease model was depressive disorder model; (3) animals were treated with scutellaria baicalensis or its active components baicalin and baicalin; (4) the data included in the literature were represented by mean and standard (SD) or can be converted to mean and SD. Exclusion criteria are as follows: (1) other types of studies (*in vitro* studies, case reports, clinical trials, reviews, abstracts or comments), (2) combination with other compounds, (3) not depressive disorder model, (5) studies with insufficient data, (6) the sample size of control group and scutellaria treatment group was less than three animals. (7) plagiarism or duplicate publication of literature.

### 2.3 Data extraction

General data, intervention measures, efficacy indicators, test results and other data of patients were independently extracted by two researchers (Ying Ma and Xun Zhou) according to a unified table and cross-checked. The following information for each study include: (1) the year of publication of the first author’s name; (2) characteristics of the animal, including species, number, sex, weight, etc.; (3) the establishment of depression model and anesthesia used in the model; (4) Characteristics of intervention, including dose and route of administration; (5) main outcome indicators and differences between groups. If the main data were lost or displayed in a graphical manner, we would contact the publishers to obtain the original data. The values in the graph were measured by digital ruler software without receiving any reply from the author.

### 2.4 Quality assessment of included studies

The methodological quality of the included studies was evaluated by independently two investigators (Ying Ma and Xun Zhou) according to the Collaborative Approach to Meta-Analysis and Review of Animal Data from Experimental Studies (CAMARADES)’s risk of bias tool (Macleod et al., 2004). The terms for quality assessment included 1) peer reviewed publication; 2) control of temperature; 3) random allocation to groups; 4) blinded induction of depression; 5) blinded assessment of behavioral outcome; 6) use of anesthetic without significant intrinsic neuroprotective activity; 7) calculation of the sample size necessary to achieve sufficient power; 8) appropriate animal model which uses animals without relevant comorbidities (aged, diabetic, or hypertensive); 9) compliance with animal welfare Regulations; 10) statement of potential conflict of interests. Any disagreements between the two researchers were resolved through negotiation or third party consensus.

### 2.5 Statistical analysis

Meta-analysis was performed using Review Manager (RevMan v5.3) software. Outcome measures were all expressed as continuous data and standardized mean difference (SMD) with 95% confidence interval (CI). If there was no statistical heterogeneity among studies (*p* ≥ 0.10, I^2^ ≤ 5%), fixed-effect model was used for analysis. Otherwise, random effect model is used to analyze. Probability value *p* < 0.05 was considered statistically significant.

## 3 Results

### 3.1 Study selection

**FIGURE 1:**
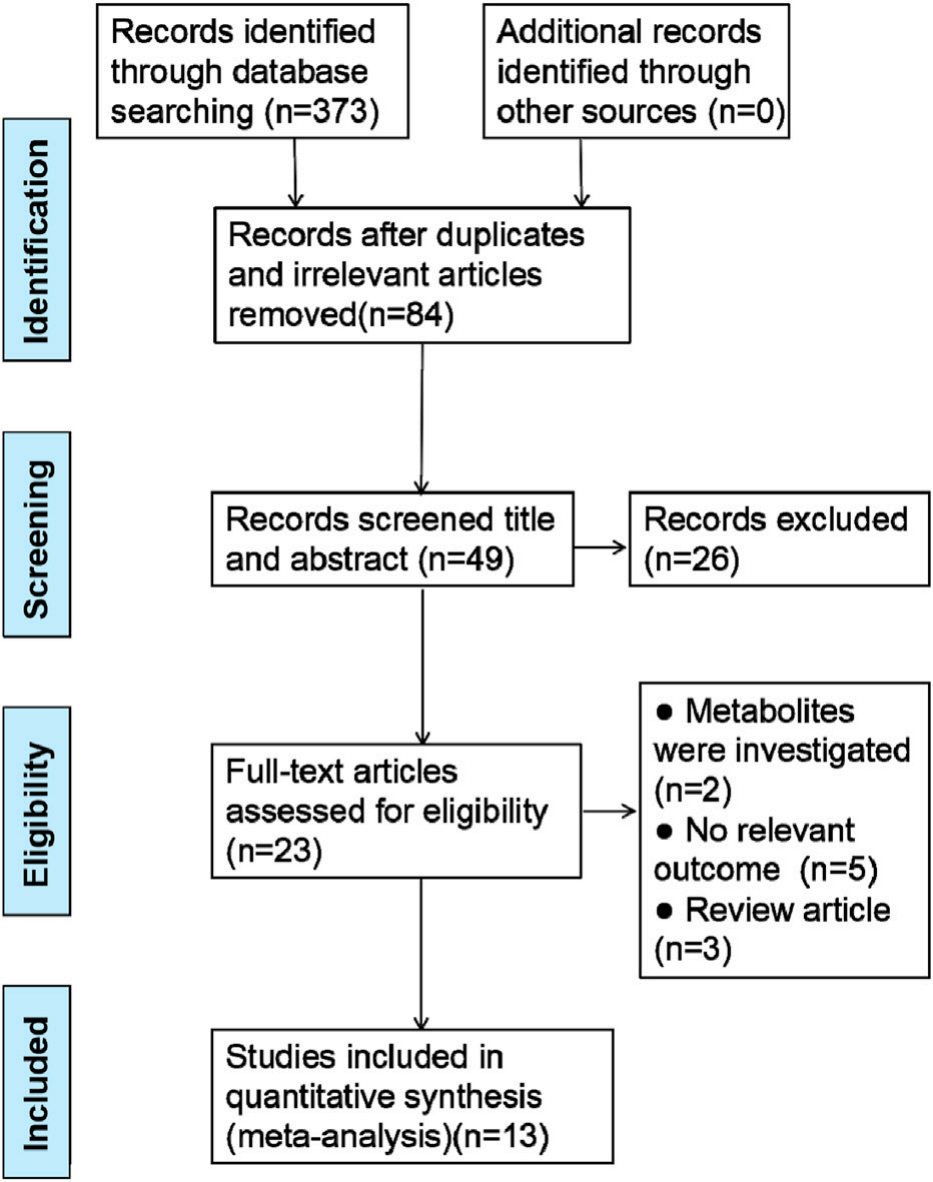
Summary of the process for identifying candidate studies.

In the initial search of databases, 373 literature were retrieved. After eliminating redundant and irrelevant articles, 84 records remained. Subsequently, the investigators screened the titles and abstracts, and 26 studies were exclude. After reviewing the full-text articles carefully, 10 studies were excluded for at least one of the following reasons: (1) metabolites were studied; (2) no relevant outcome; (3) review article. Ultimately, 13 studies were included in this meta analysis. The search strategy built on this study using the PRISMA method (Moher et al., 2009) is described in Figure 1.

### 3.2 Characteristics of included studies

The basic characteristics of the 13 studies were shown in Table 1. The meta-analysis included 270 animals (136 in the model group and 134 in the Scutellaria baicalensis treatment group) in 13 studies.

**TABLE 1:** Characteristics of studies included in systematic review of antidepressant effects of Scutellaria baicalensis.

Intervention	Depression model	Animal species (sex, n)	Weigh	Experimental group	Control group	Outcome	Intergroup difference	Time of administration	Positive drug control	Study
Baicalin	CUMS	ICR mice (male, 10/10)	22–26 g	Baicalin (25 and 50 mg/kg/d, ig)for 3W + CUMS for 6W	Saline + CUMS for 6w	(1)Behavioral test:SPT, OFT,MWM, TST, NSF; (2)Body weight↑; (3)hippocampus:a、p-ERK/ERK↑、 p-CREB/CREB↑, b、 real-time PCR: ERK mRNA↑、CREB mRNA↑, c、immunofluorescence in the CA1、CA3 and DG: p-ERK↑ 、p-CREB↑	*p* < 0.05	—	Fluoxetine, 10 mg/kg	Jia, 202 (Jia et al., 2021)
Baicalein	rotenone-induced mice model	C57BL/6J mice (male, 10/12)	20–22 g	Baicalein (300 mg/kg/d)for 4W + rotenone-induced for 2W	Saline + rotenone-induced for 2W	(1)Behavioral test:SPT↑, OFT↑,RT↑, TST↓; (2)striatum:α-synuclein↓; (3)neuroinflammation:a、midbrain:IFN-γ↓,IL-4↓,IL-12↓,IL-6↓, b、plasma: IL-1β↓, IL-4↓, IL-5↓, IL-10↓, IL-12↓; (4)monoamine neurotransmitters:a、 cortex:DA↑、 DOPAC↑、HVA↑、5-HT↑、5-HIAA↑、NE↑, b、striatum:DA↑、 DOPAC↑、HVA↑、5-HT↑, c、 brain stem:NE↑、5-HT↑; (5)BDNF/TrkB/CREB pathway in the hippocampus:PSD95↓、SYP↓, BDNF↑、TrkB↑、CREB↑, PI3K↓、Akt↓、CaMK II↓	*p* < 0.05, *p* < 0.01	—	madopar, 50 mg/kg	Zhao, 2021 (Zhao et al., 2021)
Baicalin	CORT	C57BL/6J mice (female, 10/10)	18–22 g	Baicalin (30、60 mg/kg/d)for 6W + CORT for 3W	Saline + CORT for 3W	(1)Behavioral test:SPT↑, OFT↑, FST↓, TST↓, NSF↑; (2)Hippocampal dentate gyrus:Ki-67 positive cells↑, BrdU cells↑, DCX+/NeuN+/BrdU + cells↑, DCX-/NeuN+/BrdU + cells↑; (3) PI3K/AKT/GSK3b/b-catenin pathway: p-PI3K↑, p-AKT↑, p-GSK-3b↑, b-catenin↑, PSD95↑, Synaptophysin 1↑	*p* < 0.05	30minprior to the corticosterone injection	Escitalopram, 10 mg/kg	Zhao, 2020 (Fan et al., 2020)
Baicalin	CORT, 40 mg/kg	C57BL/6 male (male, 15/15)	18–22 g	Baicalin (40、80、160 mg/kg/d)for 4W + CORT for 8W	CORT for 8W	(1)Behavioral test:FST↓, TST↓, elevated plus maze test↑; (2)DEX-induced corticosterone suppression↑; (3)nuclear GR↓, cytoplasmic pGR S203↓, cytoplasmic pGR S211↓	*p* < 0.05, *p* < 0.01	30minprior to the corticosterone injection	Fluoxetine, 18 mg/kg	Zhang, 2019a (Zhang et al., 2019a)
Baicalin	CUMS	ICR mice (male, 10/10)	23–26 g	Baicalin (60 mg/kg/d)for 3W + CUMS for 6W	Saline + CUMS for 6w	(1)Behavioral test:SPT↑, OFT↑,TST↓; (2)dendritic morphology and hippocampal neurogenesis:DCX + cells↑, dendritic intersections of neurons in the DG↑, the AOD of BrdU+/NeuN+↑; (3)Akt/FOXG1 pathway:p-Akt/Akt↑, FOXG1↑, FGF2↑	*p* < 0.01	—	Fluoxetine, 15 mg/kg	Zhang, 2019b (Zhang et al., 2019b)
Radix Scutellariae	CUMS	SD rats (male, 10/10)	190–220 g	Radix Scutellariae (500、1000 mg/kg/d)for 3W + CUMS for 6W	CUMS for 6W	(1)Behavioral test:SPT↑, OFT↑,FST↓, MWM↑; (2)hippocampus dentate gyrus:Brdu+↑, DCX+↑, NeuN+↑, Brdu+/NeuN+↑; (3)cAMP/PKA pathway:phospho-PKA↑, phosphor-CREB↑, BDNF↑	*p* < 0.05, *p* < 0.01	—	Fluoxetine, 10 mg/kg	Zhang, 2018 (Zhang et al., 2018)
Baicalein	restraint-stressed	SD rats (male, 6/6)	260–280 g	Baicalein (10, 20,40 mg/kg/d)for 2W + restraint-stressed for 2W	Saline + restraint-stressed for 2w	(1)Body weight↑; (2)Behavioral test:FST↓, OFT; (3)paraventricular nucleus:CRF↓, TH↑; (4)hippocampus and cortex:DA↑, 5-HT↑(4)BDNF mRNA↑	*p* < 0.05	30 min prior to a daily restraint stress	Fluoxetine, 10 mg/kg	Lee, 2013 (Lee et al., 2013)
Baicalein	CMS	SD rats (male, 10/10)	160–180 g	Baicalein (1, 2.4 mg/kg/d)for 3W + CMS for 3W	vehicle + restraint-stressed for 3w	(1)Behavioral test:FST↓, OFT; (2)hippocampus:p-ERK/ERK↑, BDNF↑	*p* < 0.05	1h prior to the test	imipramine, 15 mg/kg	Xiong, 2011 (Xiong et al., 2011)
Baicalein	EAP	NOD/ShiLtJ mice (male, 10/10)	16–19 g	Baicalein (100 mg/kg/d)for 1W + rat prostate antigen plus complete Freund’s adju-vant on days 0 and rat prostate antigen plus incomplete Freund’s adjuvant on days 15	Saline + EAP for 1w	(1)Behavioral test:OFT↑, EPM, FST↓, TST↓; (2) PCR: TNF-α↓, IL-1β↓, IL-6↓, IL-8↓; (3)Western blot: NF-κB p65↓, p-IκB, IκB↑	*p* < 0.05	_	_	Du, 2019 (Zhong et al., 2019)
Baicalin	CMS	Wistar rats (male, 10/10)	180–220 g	Baicalin (10, 20,40 mg/kg/d)for 5W + CMS for 5W	CMS for 5w	(1)Body weight↑; (2)Behavioral test:SPT↑; (3)serum corticosterone↓; (4)frontalcortex and hippocampus:a、 COX-2 mRNA↓, b、 COX-2 activity↓, c、PGE2↓	*p* < 0.05, *p* < 0.01	—	Fluoxetine,7 mg/kg	Li, 2013 (Liu et al., 2019)
Baicalin	CORT	ICR mice (male, 10/10)	18–22 g	Baicalin (10, 20 mg/kg/d)for 3W + CORT for 3W	vehicle + CORT for 3w	(1)Behavioral test:SPT↑, FST↓; (2)serum corticosterone↓; (3)hippocampus:a、gene expression:mRNA expression of BDNF and GR↑ 、mRNA expression of SGK1↓, b、 protein expression:BDNF↑、GR↑ 、SGK1↓	*p* < 0.05, *p* < 0.01	30 min prior to the CORT injection	Fluoxetine, 20 mg/kg	Li, 2015 (Li-Ting et al., 2019)
Baicalin	OBX	SD rat (male, 15/15)	180–220 g	Baicalin (20,40 mg/kg/d)for 2W + OBX for 2W	OBX for 2W	(1)Behavioral test:FST↓; (2)hippocampus:SOD↓, MDA↓,GSH↑; (3)Immunohistochemistry:SYP↑; (4)Apoptosis quantification:TUNEL-positive cells↓; (5) Apaf-1 caspase-3↓, caspase-9↓	*p* < 0.05, *p* < 0.01	—	amitriptyline, 10 mg/kg	Yu, 2015 (Zhang et al., 2019a)
Baicalin	CUMS	C57BL/6 mice (male, 8/8)	—	Baicalin (25,50 mg/kg/d)for 6W + CUMS for 6W	Saline + CUMS for 6w	(1)Behavioral test:SPT↑, OFT↑,TST↓,FST↓; (2)proinflammatory cytokines: IL-1b↓, IL-6↓, TNF-a↓; (3)NR2B-ERK signaling pathway: NR2B↓,CaMKII↓, P-ERK1/2↑; (4)PC12 cells:a、cell viability↑, b、 IL-1b↓、 IL-6↓、 TNF-a↓, c、 Ca2+↓ 、ROS↓, d、NR2B↓、CaMKII↓、 P-ERK1/2↑	*p* < 0.05, *p* < 0.01	—	Fluoxetine, 20 mg/kg	Zhong, 2019 (Zhang et al., 2016)

#### 3.2.1 Animals

C57BL/6 mice were used in 4 studies (Zhang et al., 2016; Zhang Kuo et al., 2019; Fan et al., 2020; Zhao et al., 2021), ICR mice were used in 3 studies (Zhang Ruyi et al., 2019; Li-Ting et al., 2019; Jia et al., 2021), and non-obese diabetic mice was used in only one study; Sprague Dawley rats were used in 4 studies (Xiong et al., 2011; Lee et al., 2013; Zhang et al., 2018; Zhang Kuo et al., 2019), and Wistar rats was used in one study (Liu et al., 2019). All but one of the studies involved males (Fan et al., 2020); The body weight of Sprague Dawley rats ranged from 160 g to 280 g, while the body weight of mice ranged from 18 g to 26 g. In the studies included in this meta-analysis, Chronic unpredictability mild stress (CUMS), corticosterone (CORT), rotenone, experimental autoimmune prostatitis (EAP), olfactory bulbectomy (OBX), and repeated restraint stress were used to construct the animal depression model. Currently, it is defined that chronic unpredictability mild stress (CUMS) is a valuable model to evaluate the etiology of depression. Among these studies, CUMS was adopted in six studies (Xiong et al., 2011; Zhang et al., 2016; Zhang et al., 2018; Zhang Ruyi et al., 2019; Liu et al., 2019; Jia et al., 2021). Long-term exposure to CORT was used in three studies (Zhang Kuo et al., 2019; Li-Ting et al., 2019; Fan et al., 2020). Rotenone (Zhao et al., 2021), EAP (Zhong et al., 2019), OBX (Zhang Kuo et al., 2019), and repeated restraint stress (Lee et al., 2013) were also applied in one studies, respectively.

#### 3.2.2 Interventions

The animals in eight studies were treated with baicalin (Zhang et al., 2016; Zhang Kuo et al., 2019; Zhang Ruyi et al., 2019; Li-Ting et al., 2019; Liu et al., 2019; Fan et al., 2020; Jia et al., 2021), four with baicalein (Xiong et al., 2011; Lee et al., 2013; Zhong et al., 2019; Zhao et al., 2021), and one with Radix Scutellariae (Zhang et al., 2018). In the 13 studies, the duration of drug administration varied, with five studies lasting 3 weeks (Xiong et al., 2011; Zhang et al., 2018; Zhang Ruyi et al., 2019; Li-Ting et al., 2019; Jia et al., 2021), two studies lasting 4 weeks (Zhang Kuo et al., 2019; Zhao et al., 2021), two studies lasting 6 weeks (Zhang et al., 2016; Fan et al., 2020), two study lasting 2 weeks (Lee et al., 2013; Zhang Kuo et al., 2019), one study lasting 1 weeks (Zhong et al., 2019), and one study lasting 5 weeks (Zhang et al., 2016). All drugs were administered intragastrically and vehicles or saline were administered to the control group in all studies.

### 3.3 Methodological quality

The assessment of the quality of these studies included in these work was conducted from CAMARADES. As shown in table 2, the quality score ranged from 6 to 7, with median of 6.615. All of the studies have been peer-reviewed and reported. All studies have reported that animals were randomly divided into groups and blinded assessment of behavioral outcome. In addition, there were no study reported blinded induction of depression.

**TABLE 2:** Quality assessment of studies included in systematic review of antidepressant effects of Scutellaria Baicalensis following modified scale of CAMARADES.

First author, year	Peer reviewed publication	Control of temperature	Random allocation to groups	Blinded induction of depression	Blinded assessment of behavioral outcome	Use of anesthetic without significant intrinsic neuroprotective activity	Calculation of the sample size necessary to achieve sufficient power	Appropriate animal model which uses animals without relevant comorbidities (aged, diabetic, or hypertensive)	Compliance with animal welfare regulations	Statement of potential conflict of interests	Quality score
Jia, 2021 (Jia et al., 2021)	1	1	1	0	1	0	0	1	1	1	7
Zhao, 2021 (Zhao et al., 2021)	1	1	1	0	1	0	0	1	1	1	7
Zhao, 2020 (Fan et al., 2020)	1	1	0	0	1	0	0	1	1	1	6
Zhang, 2019a (Zhang et al., 2019a)	1	1	1	0	1	0	0	1	1	1	7
Zhang, 2019b (Zhang et al., 2019b)	1	1	1	0	1	0	0	1	1	1	7
Zhang, 2018 (Zhang et al., 2018)	1	1	1	0	1	0	0	1	1	1	7
Lee, 2013 (Lee et al., 2013)	1	1	1	0	1	0	0	1	1	1	7
Xiong, 2011 (Xiong et al., 2011)	1	1	1	0	1	0	0	1	1	1	7
Du, 2019 (Zhong et al., 2019)	1	1	1	0	1	0	0	0	1	1	6
Li, 2013 (Liu et al., 2019)	1	1	0	0	1	0	0	1	1	1	6
Li, 2015 (Li-Ting et al., 2019)	1	1	0	0	1	0	0	1	1	1	6
Yu, 2015 (Zhang et al., 2019a)	1	1	0	0	1	0	0	1	1	1	6
Zhong, 2019 (Zhang et al., 2016)	1	1	1	0	1	0	0	1	1	1	7

### 3.4 Effects of scutellaria baicalensis on depression

#### 3.4.1 Outcome measures

The outcome measures in the 13 studies included behavioral change, physiological change, and histological analysis. For the measurment of behavioral change, six behavioral tests were commonly used, namely, sucrose preference test (SPT) (Zhang et al., 2016; Zhang et al., 2018; Zhang Ruyi et al., 2019; Li-Ting et al., 2019; Liu et al., 2019; Fan et al., 2020; Jia et al., 2021; Zhao et al., 2021), open field test (OFT) (Xiong et al., 2011; Lee et al., 2013; Zhang et al., 2016; Zhang et al., 2018; Zhang Ruyi et al., 2019; Zhong et al., 2019; Fan et al., 2020; Jia et al., 2021; Zhao et al., 2021), morris water maze test (Zhang et al., 2018; Jia et al., 2021), tail suspension test (Zhang et al., 2016; Zhang Kuo et al., 2019; Zhang Ruyi et al., 2019; Zhong et al., 2019; Fan et al., 2020; Jia et al., 2021; Zhao et al., 2021), novelty suppressed feeding test (NSFT) (Fan et al., 2020; Jia et al., 2021), forced swimming test (Xiong et al., 2011; Lee et al., 2013; Zhang et al., 2016; Zhang Kuo et al., 2019; Li-Ting et al., 2019; Zhong et al., 2019; Fan et al., 2020). For the determination of physiological change, body weight, sucrose intake and sleep were assessed (Liu et al., 2019; Jia et al., 2021). For the detection of histological analysis, all of the studies have all focused on the hippocampus, and some of these studies also assessed a broader range of regions, such as striatum (Zhao et al., 2021), paraventrnucleus (Lee et al., 2013), cortex (Lee et al., 2013; Liu et al., 2019; Zhao et al., 2021), midbrain (Zhao et al., 2021) or brain stem (Zhao et al., 2021).

#### 3.4.2 Effects of scutellaria baicalensis on depression by SPT analysis

**FIGURE 2:**
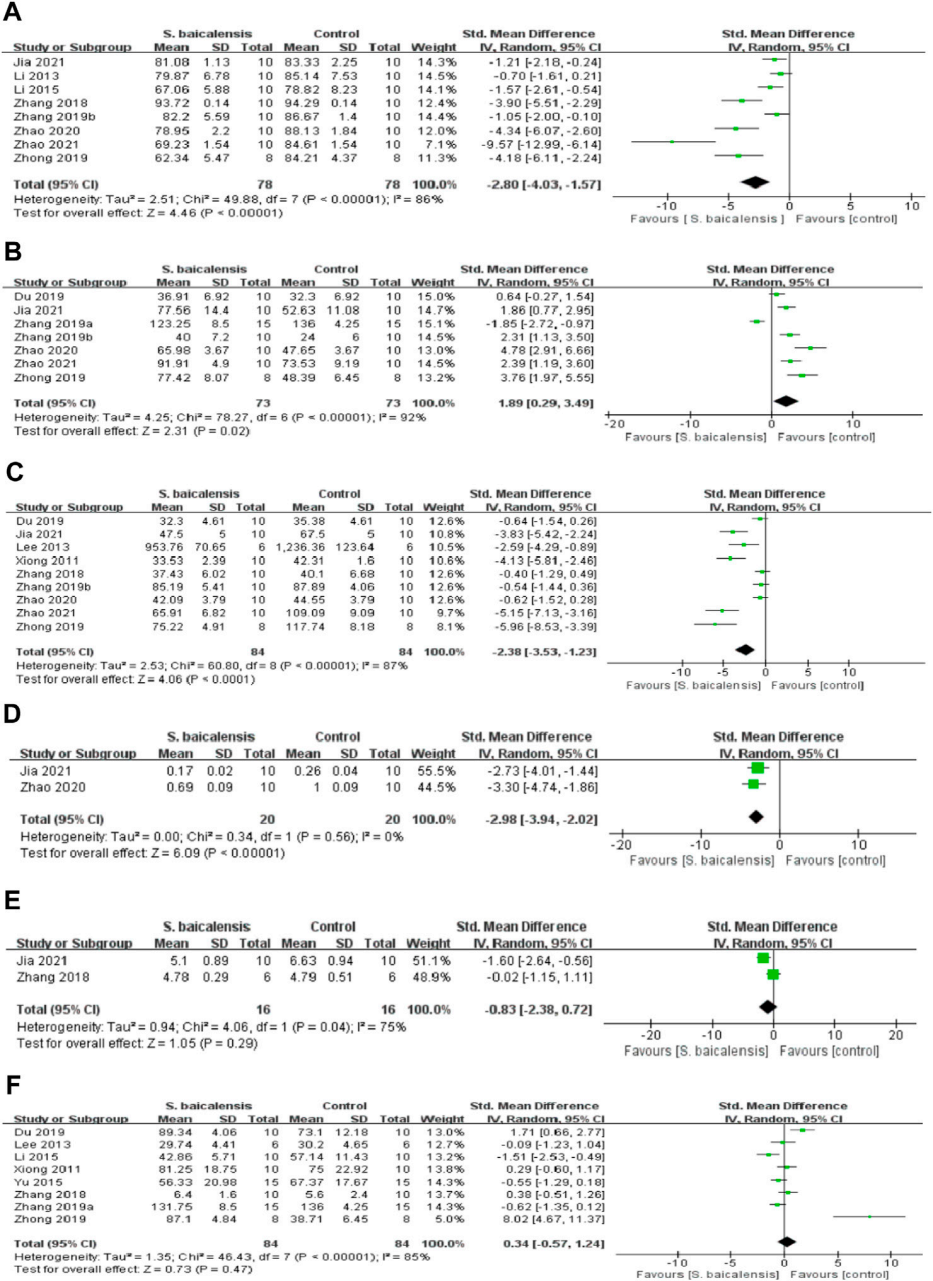
Forest plot of studies investigating the effect of Scutellaria baicalensis on animal behavior. The protective effects of Scutellaria baicalensis on animal behavior by **(A)** SPT, **(B)** TST, **(C)** OFT, **(D)** NSFT, **(E)** MWM, and **(F)** FST analysis.

A total of eight studies (Zhang et al., 2016; Zhang et al., 2018; Zhang Ruyi et al., 2019; Li-Ting et al., 2019; Liu et al., 2019; Fan et al., 2020; Jia et al., 2021; Zhao et al., 2021)compared the differences in SPT before and after treatment with 158 animals, including 78 in the experimental group and 80 in the control group. Due to significant heterogeneity between studies (*p* < 0.00001, I^2^ = 90%), the random-effects model was adopted. The difference between the two groups was statistically significant [SMD = −2.80, 95%CI(-4.03, -1.57), *p* < 0.01], suggesting that scutellaria baicalensis could significantly enhance the sucrose preference rate in depressed animals, as shown in Figure 2A.

#### 3.4.3 Effects of scutellaria baicalensis on depression by TST analysis

The analysis of TST was applied in seven studies (Zhang et al., 2016; Zhang Kuo et al., 2019; Zhang Ruyi et al., 2019; Zhong et al., 2019; Fan et al., 2020; Jia et al., 2021; Zhao et al., 2021) that had a total sample size of 148, of which 73 animals received Scutellaria baicalensis and 75 received a vehicle or saline treatment, while the analysis of FST covered eight studies (Xiong et al., 2011; Lee et al., 2013; Zhang et al., 2016; Zhang Kuo et al., 2019; Li-Ting et al., 2019; Zhong et al., 2019; Fan et al., 2020)with 168 animals, 84 in the experimental group and 84 in the control group. There were significant heterogeneity among the studies (*p* < 0.00001, I^2^ = 92%; *p* < 0.00001, I^2^ = 85%), the random-effect model was adopted. Results as shown in Figures 2B, F, immobility time was dramatically reduced in the Scutellaria baicalensis group *versus* control group, the difference was no statistically significant ([SMD = 1.89, 95%CI(0.29, 3.49), *p* = 0.02]; [SMD = 0.34, 95%CI(-0.57, 1.24), *p* = 0.47].)

#### 3.4.4 Effects of scutellaria baicalensis on depression by OFT analysis

In OFT, meta-analysis of nine studies (Xiong et al., 2011; Lee et al., 2013; Zhang et al., 2016; Zhang et al., 2018; Zhang Ruyi et al., 2019; Zhong et al., 2019; Fan et al., 2020; Jia et al., 2021; Zhao et al., 2021)had a total sample size of 170, of which 84 animals received Scutellaria baicalensis and 86 received a vehicle or saline treatment. There was Statistically significant between groups in the number of crossings and rearing, indicating that Scutellaria baicalensis treatment can ameliorate the frequency of crossing and rearing compared with the control group [SMD = −2.38, 95%CI(-3.53, -1.23), *p* < 0.01], as shown in Figure 2C.

#### 3.4.5 Effects of scutellaria baicalensis on depression by NFST analysis

In the NSFT experiment (Fan et al., 2020; Jia et al., 2021), food consumption was increased [SMD = −2.98, 95%CI(-3.94, -2.02), *p* < 0.01 in Scutellaria baicalensis group compared to control group. In contrast, there was no statistically significant differences in the MWM (Zhang et al., 2018; Jia et al., 2021), implying that Scutellaria baicalensis administration did not affect the times of platform crossings and seconds spent in the target quarter [SMD = −0.83, 95%CI(-2.38,0.72), *p* = 0.29; Figure 2E].

### 3.5 Underlying mechanisms

Most of the included literature is based on studies of the neuroprotective effects of Scutellaria baicalensis on depression. Brain-derived neutrophic factor (BDNF) (Xiong et al., 2011; Lee et al., 2013; Zhang et al., 2018; Li-Ting et al., 2019; Jia et al., 2021; Zhao et al., 2021), extracellular-signal-regulated kinase (ERK) phosphorylation (Xiong et al., 2011; Zhang et al., 2016; Jia et al., 2021), cAMP response element-binding protein (CREB) phosphorylation (Zhang et al., 2018; Jia et al., 2021; Zhao et al., 2021), tropomyosin-related kinase B (TrkB) phosphorylation (Zhao et al., 2021), protein kinase A (PKA) phosphorylation (Zhang et al., 2018), phosphatidylinositol 3-kinasep (PI3K) phosphorylation (Fan et al., 2020), Glycogen synthase kinase-3 beta (GSK3b)phosphorylation (Fan et al., 2020), Protein Kinase B (AKT) phosphorylation (Zhang Kuo et al., 2019; Fan et al., 2020), NF-kB p65 phosphorylation (Zhong et al., 2019), inhibitor of kB (IkB) phosphorylation (Zhong et al., 2019), Fibroblast growth factor (FGF) (Zhang Ruyi et al., 2019), Forkhead box G1 (FOXG1) (Zhang Ruyi et al., 2019), Serum/Glucocorticoid Regulated Kinase 1(SGK-1) (Li-Ting et al., 2019), N-methyl-D-aspartate receptor 2B (NR2B) (Zhang et al., 2016), and CaMKII (Zhang et al., 2016) were studied. Mechanistically, by acting as partial, subtype-selective GABAA receptor ligands, scutellaria baicalensis and its bioactive ingredients (baicalin and baicalein) foster the interaction of GABAA receptors with TrkB to potentiate GABA-induced signaling. By increasing cAMP/pERK and PI3K/pAKT signaling, they promote the synthesis of neurotrophic factors (BDNF and NGF) as well as neurogenesis.

As far as monoamine neurotransmitters is concerned, dopamine (DA) (Lee et al., 2013; Zhao et al., 2021), 3,4-dihydroxyphenylacetic acid (DOPAC) (Lee et al., 2013; Zhao et al., 2021), homovanillic acid (HVA) (Lee et al., 2013; Zhao et al., 2021) serotonin (5-HT) (Lee et al., 2013; Zhao et al., 2021), 5-hydroxyindole-acetic acid (5-HIAA) (Zhao et al., 2021), and noradrenaline (NE) (Zhao et al., 2021) were assessed. In addition, serum Corticosterone (Zhang Kuo et al., 2019; Li-Ting et al., 2019; Liu et al., 2019), glucocorticoid receptor phosphorylation in hypothalamus (Zhang Kuo et al., 2019; Li-Ting et al., 2019), corticotrophin-releasing factor (CRF)in hypo-thalamic (Lee et al., 2013), and tyrosine hydroxylase (TH)in hypo-thalamic (Lee et al., 2013) were evaluated. BrdU^+^ (Zhang et al., 2018; Zhang Ruyi et al., 2019; Fan et al., 2020), NeuN^+^ (Zhang et al., 2018; Zhang Ruyi et al., 2019; Fan et al., 2020) and DCX^+^ (Zhang et al., 2018; Fan et al., 2020) in DG; SLC6A4 [473], IDO (Zhong et al., 2019) GFAP (for astrocytes) (Zhong et al., 2019), Iba1 (for microglia) (Zhong et al., 2019) in the CA1, CA3, andDG (Zhong et al., 2019); taurine (Tau)/totalcreatine (tCr,creatine + phosphocreatine), glutamate + glutamine (Glx)/tCr in the hippocampus (Zhong et al., 2019); and TUNEL-positive cells in the hippocampus (Zhang Kuo et al., 2019) were also examined. Additionally, α-synuclein (Zhao et al., 2021), PSD95 (Fan et al., 2020; Zhao et al., 2021), and SYP (Zhang Kuo et al., 2019; Fan et al., 2020; Zhao et al., 2021) were also studied. Actually, By acting as MAO A/B inhibitors, scutellaria baicalensis induce monoamine, and mostly DA release.

To determine the anti-inflammatory effects of Scutellaria baicalensis during depression, IFN-γ, TNF-α, IL-1β, IL-2, IL-4, IL-5, IL-6, IL-10, IL-12 in plasma (Zhao et al., 2021) and TNF-α, IL-1β, IL-6, IL-8, IL-18 in the hippocampus (Zhong et al., 2019) were studied respectively. Furthermore, inflammatory factors such as IL-1β, IL-6, and TNF-α were measured both in the serum and in the hippocampus (Zhang et al., 2016). In terms of chronic oxidative stress and apoptosis, COX-2 in the frontal cortex and hippocampus (Liu et al., 2019), PGE 2 in the frontalcortex and hippocampus (Liu et al., 2019), SOD (Zhang Kuo et al., 2019), MDA (Zhang Kuo et al., 2019), GSH (Zhang Kuo et al., 2019), Apaf-1 caspase-3 (Zhang Kuo et al., 2019), caspase-9 (Zhang Kuo et al., 2019)in the hippocampus, [Ca ^2+^ ] (Zhang et al., 2016) and ROS (Zhang et al., 2016) were mainly appraised.

## 4 Discussion

Depression is a common mental disease associated with high morbidity and huge social burden. Numerous preclinical studies have shown that Scutellaria baicalensis and its active constituents are effective for depression. The aim of this study is to systematically review the roles of scutellaria baicalensis in depression and summarize the possible mechanism. To our knowledge, it is a first systematic review and meta-analysis of preclinical studies on the efficacy of Scutellaria baicalensis and its main components in animal depression model. The results indicated that Scutellaria baicalensis can remarkably safeguard against depression evidenced by improved behavioral changes. And this activities were associated with the regulation of Scutellaria baicalensis on inflammatory responses, oxidative stress, apoptosis, and neurotransmitters production via modulating TrkB-BDNF, PI3K-AKT, MAPK and NF-κB pathways.

In this work, 13 studies were included to assess the efficacy of scutellaria baicalensis on depression. Chronic unpredictability mild stress, corticosterone, rotenone-induced depression model, experimental autoimmune prostatitis (EAP)-induced depression mice model, olfactory bulbectomy (OBX) depression mice model and repeated restraint stress-induced depression rat model were used to study the antidepressant effect of Scutellaria baicalensis, while sucrose preference test (SPT), open field test (OFT), Morris water maze test (MWM), tail suspension test (TST), novelty suppressed feeding test (NSFT), and forced swimming test (FST) were adopted to evaluate the efficacy.

So far, the anti-depressive actvities of scutellaria baicalensis are welly studied, and the commonly used animal models are CUMS (Xiong et al., 2011; Zhang et al., 2016; Zhang et al., 2018; Zhang Ruyi et al., 2019; Liu et al., 2019; Jia et al., 2021)and CORT (Zhang Kuo et al., 2019; Li-Ting et al., 2019; Fan et al., 2020), etc., and one of the criteria to evaluate the success of depression model and the efficacy of anti-depression is behavioral test. Sucrose preference test for evaluating degree of pleasure lack of mice, open field test reflect the spontaneous activity in mice and explore the behavior, forced swimming test and tail suspension test reflects the behavior of the mice desperation, reaction ability of learning and memory in mice water maze experiment, while novelty suppressed feeding test, the variation of the lack of animal euphoria (Lu et al., 2016). In a mouse model of chronic cort-induced depression, baicalin significantly ameliorates behavior change by reducing the time spent in the central area of the open field test and the time spent in the cross maze test with open arms, and increasing the immobile time in the tail suspension test and forced swimming test. (Zhang et al., 2016; Zhang Kuo et al., 2019). On the other hand, Scutellaria baicalensis alleviated depression-like behavior by increasing sucrose consumption and reducing the immobile time of tail suspension and forced swimming tests in mild stress chronic mouse model (CUMS) (Li-Ting et al., 2019; Liu et al., 2019; Zhong et al., 2019).

The anti-depressive mechanism of scutellaria baicalensis is still not fully understand. Scutellaria baicalensis and its active components have a wide range of antidepressive effects. This review shows that most studies have focused on neural protection by measuring BDNF, ERK, CREB, TrkB, PI3K, GSK3B, AKT, NF-KB P65, IkB and their phosphorylation (Xiong et al., 2011; Lee et al., 2013; Zhang et al., 2018; Zhang Kuo et al., 2019; Li-Ting et al., 2019; Zhong et al., 2019; Fan et al., 2020; Jia et al., 2021; Zhao et al., 2021). Neuroinflammation was concerned by detecting inflammatory cytokines IL-1β, IL-6 and TNF-α in serum and/or hippocampus (Zhang et al., 2016; Zhong et al., 2019; Zhao et al., 2021). Monoamine neurotransmitters including dopamine, 3, 4-dihydroxyphenylacetic acid, hypervanilic acid, serotonin (5-HT), 5-hydroxyindoleacetic acid, and norepinephrine were evaluated (Lee et al., 2013; Zhao et al., 2021). Oxidative stress and apoptosis were studied by measuring COX-2,SOD, MDA, GSH, APAF1 Caspase-3, Caspase-9, [Ca ^2+^]and ROS levels in frontal lobe and hippocampus (Zhang et al., 2016; Zhang Kuo et al., 2019; Liu et al., 2019). Moreover, protein expressions of the presynaptic marker (synaptophysin1) and the postsynaptic marker (PSD95) and so on were studied following baicalin treatment (Fan et al., 2020; Zhao et al., 2021). The included studies mostly confirmed that scutellaria baicalensis regulated monoamine neurotransmitter levels and inflammatory factors. In addition, scutellaria baicalensis and its active components can also regulate oxidative stress, apoptosis, and synaptic dysfunction.

These results resembled theoretical study. Studies have reported that scutellaria baicalensis may induce the release of monoamine (DA) and enhance GABA-induced signal transduction, thereby increasing cAMP/pERK and PI3K/pAKT signals, and promoting the synthesis and neurogenesis of neurotrophic factors (BDNF and NGF) (Im Heh-In et al., 2005; Mu Xin et al., 2011). Secondly, scutellaria baicalensis plays an anti-inflammatory role by decreasing the levels of inducible nitric oxide synthase, NF-κB and pro-inflammatory cytokines TNF-α, IL-6 and IL-1β (Ma et al., 2015). It is reported that scutellaria baicalensis can also improve mitochondrial membrane potential depolarization and ATP production, and increase AMPK to enhance mitochondrial autophagy and mitochondrial biogenesis, thus playing a protective role in mitochondria (Zhang et al., 2017). It also lowers levels of reactive oxygen species and nitrogen species, increases superoxide dismutase, glutathione, glutathione peroxidase and catalase activities, heat shock protein 70, heme oxygenase-1 and thioredoxin levels, and ultimately reduces lipid peroxidation. The content of malondialdehyde and lipoxygenase are decreased by Scutellaria baicalensis. It also inhibited p-P38, Bax/Bcl-2 ratio, caspase 3, caspase 6, caspase 9, and cytochrome C release, thereby inhibiting apoptosis (Liu et al., 2012; Wang et al., 2013; Li et al., 2019).

This study was screened strictly in accordance with the inclusion criteria, exclusion criteria and literature quality scoring criteria, but there may still be the following limitations that may affect the accuracy of the study: First, the databases searched in this review were all in English, so there may be some deviations; Second, the methodological quality of the included studies was moderate, with none of the studies reporting the blinded induction of depression and the sample size needed to calculate it to obtain sufficient power; Third, the lack of negative studies may lead to overestimation of the true role of Scutellaria baicalensis. Fourthly, in the included studies, there were significant differences in depression modeling method and time, dosage and treatment time of scutellaria baicalensis.

## 5 Conclusion

In this preclinical systematic review, scutellaria baicalum can improve the symptoms of anhedonia, reduce the degree of behavioral despair, improve the cognitive ability of mice and play an anti-depressive effect in experimental depression. The mechanism mainly includes antioxidant, anti-inflammatory, neurotransmitter regulation, and inhibition of apoptosis. Therefore, scutellaria baicalensis may be a candidate for further clinical trials of depression.

## Data Availability

The original contributions presented in the study are included in the article/Supplementary material, further inquiries can be directed to the corresponding authors.
